# *In vitro* fertilization program in white rhinoceros

**DOI:** 10.1530/REP-23-0087

**Published:** 2023-10-25

**Authors:** Thomas Bernd Hildebrandt, Susanne Holtze, Silvia Colleoni, Robert Hermes, Jan Stejskal, Isaac Lekolool, David Ndeereh, Patrick Omondi, Linus Kariuki, Domnic Mijele, Samuel Mutisya, Stephen Ngulu, Sebastian Diecke, Katsuhiko Hayashi, Giovanna Lazzari, Barbara de Mori, Pierfrancesco Biasetti, Alessandra Quaggio, Cesare Galli, Frank Goeritz

**Affiliations:** 1 Leibniz Institute for Zoo and Wildlife Research (IZW) in the Forschungsverbund Berlin eV, Reproduction Management, Alfred-Kowalke-Straße, Berlin, Germany; 2 Freie Universitat Berlin, Veterinary Medicine, Berlin, Germany; 3 AVANTEA, Laboratorio di Tecnologie della Riproduzione, Lombardy, Cremona, Italy; 4 ZOO Dvůr Králové, Communication and International Projects, Štefánikova, Dvůr Králové nad Labem, Czech Republic; 5 Kenya Wildlife Service, Veterinary and Capture Services, Nairobi, Kenya; 6 Wildlife Training and Research Institute, Nakuru County, Naivasha, Kenya; 7 Ol Pejeta Conservancy, Conservation Laikipia, Nanyuki, Kenya; 8 Max-Delbrück-Center for Molecular Medicine in the Helmholtz Association (MDC), Technology Platform Pluripotent Stem Cells, Berlin, Germany; 9 Department of Stem Cell Biology and Medicine, Kyushu University, Maidashi, Higashiku, Fukuoka, Japan; 10 Graduate School of Medicine, Osaka University, Suita, Osaka, Japan; 11 Department of Comparative Biomedicine and Food Science, Università degli Studi di Padova, Italy; 12 Universita degli Studi di Padova, Ethics Laboratory for Veterinary Medicine, Conservation, and Animal Welfare, Veneto, Padova, Italy; 13 Fondazione Avantea, Riproduzione Cremona, Lombardy, Cremona, Italy

## Abstract

**In brief:**

To save endangered rhinoceros species, assisted reproductive technologies are warranted. We here report *in vitro* blastocyst generation of the Near-Threatened Southern white rhinoceros and, for the first time, also of the technically Extinct Northern white rhinoceros.

**Abstract:**

The Anthropocene is marked by a dramatic biodiversity decline, particularly affecting the family Rhinocerotidae. Three of five extant species are listed as Critically Endangered (Sumatran, Javan, black rhinoceros), one as Vulnerable (Indian rhinoceros), and only one white rhino (WR) subspecies, the Southern white rhinoceros (SWR), after more than a century of successful protection is currently classified as Near Threatened by the IUCN, while numbers again are declining. Conversely, in 2008, the SWR’s northern counterpart and second WR subspecies, the Northern white rhinoceros (NWR), was declared extinct in the wild. Safeguarding these vanishing keystone species urgently requires new reproductive strategies. We here assess one such strategy, the novel *in vitro* fertilization program in SWR and – for the first-time NWR – regarding health effects, donor-related, and procedural factors. Over the past 8 years, we performed 65 procedures in 22 white rhinoceros females (20 SWR and 2 NWR) comprising hormonal ovarian stimulation, ovum pick-up (OPU),* in vitro* oocyte maturation, fertilization, embryo culture, and blastocyst cryopreservation, at an efficiency of 1.0 ± 1.3 blastocysts per OPU, generating 22 NWR, 19 SWR and 10 SWR/NWR hybrid blastocysts for the future generation of live offspring.

## Introduction

Currently, rhinoceroses are among the most threatened megavertebrate species. Recent extinctions comprise the Vietnamese Javan (*Rhinoceros sondaicusannamiticus* (Brook *et al.* 2014) and the Western black rhinoceros (*Dicerosbicornislongipes* (Emslie 2020*a*) in 2010 and 2011), respectively. The remaining population of the critically endangered Indonesian subspecies of the Javan rhinoceros (*Rhinoceros sondaicussondaicus*) has dwindled to ~75 individuals – and both Sumatran rhinoceros subspecies (*Dicerorhinussumatrensissumatrensis and D. s. harrisoni*) to 46–66 individuals (Ellis & Talukdar 2020*a*,*b*). The black rhinoceros (*Dicerosbicornis*) is also listed as critically endangered (Emslie 2020*b*). Of the two white rhino (WR) subspecies – Southern (SWR; *Ceratotheriumsimumsimum*) and Northern white rhinoceros (NWR; *C. s. cottoni*) – the latter was recently reduced to two infertile females, destined to be the next in line for annihilation (Emslie 2020*c*, Hildebrandt *et al.* 2021*a*,*b*).

In this context and in the light of barely self-sustaining captive rhinoceros populations (Swaisgood *et al.* 2006), assisted reproductive technologies (ARTs) are indispensable for safeguarding these keystone species, which play crucial roles in their complex ecosystems. Poaching for rhinoceros horn – partly for financing armed conflicts – and habitat loss are the main drivers of the human-made extinction of these charismatic landscape architects (Brook *et al.* 2014, Martin 2019).

To a total of 23 wild-caught NWR held in captivity since 1949, only six calves were born – one NWR/SWR hybrid, one stillborn, one male, and three females – among them the last two remaining NWR Najin and Fatu. All births occurred at Dvůr Králové Zoo in Czech Republic from four founder individuals. Although past zoo breeding was insufficient to maintain a viable captive population, the only two currently living NWR testify to both responsibility and the potential of zoos for biodiversity protection (Che-Castaldo *et al.* 2018, Bolam *et al.* 2021).

The ART procedures developed for different rhinoceros species comprise semen collection and cryopreservation (Schaffer *et al.* 1990, Hildebrandt *et al.* 2000, Roth *et al.* 2005, Hermes *et al.* 2018, Rickard *et al.* 2022) as well as artificial insemination (AI) (Hildebrandt *et al.* 2000, 2007, Hermes *et al.* 2009, Stoops *et al.* 2016). For SWR, recently, ovum pick-up (OPU) combined with *in vitro* oocyte maturation, intracytoplasmic sperm injection (ICSI), embryo culture, and embryo cryopreservation have been implemented for successful *in vitro* blastocyst and embryonic stem cell (ESC) generation (Hildebrandt *et al.* 2018*a*).

In NWR, OPU-derived natural embryos seem the only realistic route to generate calves in due time to allow for transfer of species-specific knowledge, vocalizations, and behaviors from the last two adults to the next generation. Here, we report the first successful application of *in vitro* fertilization (IVF) procedures in NWR.

The embryos are the basis for generating new individuals in future embryo transfers (ETs) to surrogate recipients of the same, or in case of the NWR, a closely related taxon, the SWR. Alternative, but more challenging and futuristic approaches might be using an artificial womb (Partridge *et al.* 2017) or a more remotely related taxon, which requires chimera construction, i.e. transferring the inner cell mass (ICM) of a rhinoceros embryo into the trophoblast of the recipient taxon (Loi *et al.* 2007, 2018, Saragusty *et al.* 2016).

Further novel advanced ART protocols will be needed in the long run, complementing the available portfolio to successfully restore the necessary genetic diversity when population size has decreased too far (as in the NWR). Stem cell-based approaches such as generation of embryonic (Hildebrandt *et al.* 2018*a*) and induced pluripotent stem cells (iPSC; Ben-Nun *et al.* 2011, Korody *et al.* 2021, Zywitza *et al.* 2022) for *in vitro* gametogenesis (Hayashi *et al.* 2022) and ultimately embryo production will be essential (Saragusty *et al.* 2016, Hildebrandt *et al.* 2018*a*). Embryos may be generated by either combining iPSC-derived artificial gametes of one sex with natural gametes of the opposite sex, by exclusively using artificial gametes or by embryo construction from iPSC-derived germ cell layers (Liu *et al.* 2021, Sozen *et al.* 2021).

In light of the importance of rhinoceros OPU and *in vitro* blastocyst generation for conservation, we here review our experience gained during 65 OPU procedures in 20 SWR and, for the first time, in 2 NWR over the past 8 years. This ambitious B*io*R*escue* project aims at optimizing genetic management in the European captive SWR population, developing international ET-based gene exchange programs, saving the NWR from extinction, and creating a blueprint for the rescue of further endangered species.

## Materials and methods

### Animals and health status

This study was approved by the Internal Committee for Ethics and Animal Welfare of the Leibniz-IZW (#2015-03-03). Between June 2015 and July 2022, 22 WR (20 SWR and 2 NWR) were submitted to a total of 65 OPUs in 15 facilities – 14 European zoological institutions and 1 Kenyan Wildlife Conservancy. OPU was performed once in 9 females, and repeatedly at an interval ≥3 months: twice (*n = *2), 3 (*n = *4), 4 times (*n = *2), 5 times (*n = *3), 7 times (*n = *1), and 10 times (*n = *1).

In agreement with the European Association of Zoos and Aquaria (EAZA) Rhinoceros Taxon Advisory Group (TAG) chair and European Endangered Species Programme (EEP) coordinator for White Rhinoceroses, only animals with a history of impaired natural breeding or transport restriction due to infectious disease were included in the European IVF program. The youngest and one of the few entirely reproductively healthy females in the study cohort was included due to COVID-19 and Brexit-related delay in arrival of a breeding bull to the destined facility. The 22 females in this study comprised females at the end of their reproductive lifespan (which may be accelerated in nonbreeding females) (*n = *5), females with repeated conception failure despite mating (*n = *7), temporary lack of access to a suitable breeding bull (*n = *4), failure to mate (*n = *2), and females recommended to cease natural breeding, e.g. due to positive tuberculosis (TB)-test status (*n = *4). Two procedures were discontinued upon unexpected pregnancy detection. Reproductive health status was assessed at the initial ultrasound examination during the OPU procedure.

### Ovarian stimulation

Before each OPU, the donor female received several consecutive gonadotropin-releasing hormone (GnRH) slow release injections (3 mL Histrelin® BioRelease, 0.5 mg/mL, Bet Pharm LLC, USA) intramuscularly every other day either by hand injection or via dart application. Injections were repeated either 3 (*n = *63) or 4 (*n = *1) times; one OPU was undertaken without preceding hormonal stimulation in a female that had previously undergone four hormonally stimulated OPUs. The OPU was performed 2 (*n = *3), 3 (*n = *56), or 4 (*n = *5) days after administering the last GnRH injection, respectively. No prestimulation ultrasonographic assessments were performed. Therefore, ovarian activity or estrous cycle state at treatment onset was usually unknown. If mating was observed, to optimally stimulate follicular growth, the first hormone injection was scheduled 20 days later, thus in the follicular phase, to avoid hormonal interference of the active corpus luteum (CL) during the luteal phase.

### Anesthesia

OPU procedures were performed under full anesthesia in sternal or lateral recumbency inside a confined area (Walzer *et al.* 2010) using a combination of butorphanol tartrate (50 mg/animal), detomidine hydrochloride (30 mg/animal), midazolam hydrochloride (20 mg/animal), and ketamine hydrochloride (200 mg/animal) for induction, administered either intramuscularly via dart or by hand injection, depending on the individual animal’s training status. For maintenance, once the patient was approachable, 500 mg ketamine hydrochloride, 50 mg butorphanol tartrate, 10 mg detomidine hydrochloride, and 5 mg midazolam hydrochloride diluted in 500 mL 0.9% sodium chloride were administered into the ear vein within 60–90 min (CIR = 15–35 mL/min). After OPU completion, anesthesia was reversed using atipamezole hydrochloride (70–100 mg/animal) and naltrexone hydrochloride (60–80 mg/animal) with a time to standing of 2–5 min.

### OPU procedure

To avoid fecal contamination in the area of transrectal ovarian puncture and to minimize the associated risk of infection, the intestine was emptied as far as possible from the anus upward to the caudal parts of the colon. Rectum and adjacent colon were then cleaned up to 2 m with lukewarm water and a mild mucosal disinfectant (Octenisept®). The patented OPU device (U.S.: # 10,779,859; Europe: # EP 3369397; ARIPO: # AP/P/2018/010558; Republic of South Africa: # 2018/01416) of 150 cm length was introduced into the rectum using sterile lubricant (ReproJelly, Minitüb GmbH, Germany).

The procedure was performed under ultrasound guidance (Voluson I, GE Healthcare, Germany) with a probe (RNA5-9-RS, 5.0–9.0 MHz) embedded in a 3D-printed case with integrated needle guide. A customized double-lumen needle with inner and outer diameters of 1.4 mm and 2.1 mm, respectively, was placed adjoining the ovary, approximately 120–140 cm cranial to the anus. The needle can be advanced straight for up to 80 mm into the ovarian follicles at an angle of 85° to the axis of the OPU device. Follicles were punctured, aspirated, and flushed, i.e. refilled to original size up to ten times with a flushing media (Euroflush, IMV, France) containing 20 IU/mL heparin (Ratiopharm, Germany). Up to ten follicles were flushed before repositioning the OPU device and repuncturing the intestinal wall for harvesting the next follicle cohort. After completion of the OPU, local antibiotics (3 intra-uterine tablets, 2400 mg amoxicillin trihydrate) were deposited into the colon. Technical details of the equipment were frequently adjusted to overcome encountered problems. Ultrasound data of 55 procedures were recorded and average, maximal, and minimal diameters of all follicles were measured. In two individuals with unilateral ovarian tumors (1 NWR, 1 SWR) and in six further cases (1 NWR, 5 SWR), oocytes were collected unilaterally due to technical or anatomical difficulties or the absence of follicles on one of the ovaries.

### Oocyte retrieval and shipping

The collected fluid was filtered using a 70 µm nylon mesh (Cell Strainer, Corning Optical Communications GmbH, Germany), later replaced by an embryo filter-system (EmSafe, Minitube GmbH, Germany). Oocytes were searched under a stereo microscope (Stemi 508, Carl Zeiss Microscopy GmbH; Nikon SMZ1270, Nikon Europe B.V.). After washing in tissue culture plates (Falcon, Germany) with holding solution (H-SOF, Hepes-Synthetic Oviductal Fluid), oocytes were individually transferred into sterile 2 mL H-SOF-containing Nalgene® cryogenic vials (Merck KGaA, Germany). Transport to Avantea laboratory (Cremona, Italy) was realized inside a portable battery-driven incubator (Micro Q Technologies LLC, USA) at a constant temperature of 22°C equipped with a temperature logger (TempU S3, Kronsguard GmbH, Germany). To avoid delays with overnight courier services, especially during the COVID-19 pandemic, a team member often carried out the transport in person (≤48 h). Cumulus–oocyte complexes (COCs) and cryopreserved semen transport complied with national and international laws.

### Sperm

For ICSI, the sperm of 7 bulls (4 NWR, 3 SWR) was employed, previously obtained by electroejaculation (Seager model 14, Dalzell USA Medical Systems, USA), cryopreserved in 0.5 mL straws, and stored in liquid nitrogen (lN_2_) was employed.

Between 2003 and 2014, 300 mL NWR sperm of four bulls (Saut, Suni, Angalifu, and Sudan) were cryopreserved; samples were tested in the standardized xenogenic porcine oocyte cleavage assay for the ability of spermatozoa to form pronuclei after ICSI and initiate cleavage (Hildebrandt *et al.* 2018*a*).

### *In vitro* maturation, ICSI, and embryo culture

At Avantea laboratory, oocytes were transferred into a DMEM-F12-based maturation media (Colleoni *et al.* 2007). They were cultured at 37.5°C in a humidified atmosphere containing 5% CO_2_ in air for 36–40 h, before hyaluronidase treatment and mechanical denuding of cumulus cells. The maturation protocol, initially designed for equine oocytes, was considered the most suitable for the rhinoceros due to their phylogenetic proximity with the horse. It was optimized lowering the temperature to 37.5°C on the basis of the white rhinoceros physiological body temperature. Due to the high value of these biological samples, all the collected oocytes were always subjected to maturation, without any kind of morphological selection. Therefore, cultured oocytes were heterogeneous regarding dimensions, number of cumulus layers, and ooplasm appearance.

Frozen-thawed semen was centrifuged on a discontinuous density gradient (RediGrad 90–45%) to recover viable spermatozoa and diluted in mSOF IVF medium (Lazzari *et al.* 2002). Just before ICSI, semen was diluted in PVP 10% in H-SOF.

ICSI was performed on a heated stage of an inverted Nikon Eclipse TE300 microscope equipped with a piezoelectric micromanipulation-driven micropipette (Eppendorf, Hamburg, Germany). Single motile spermatozoa with normal morphology were immobilized by piezo pulses. They were then injected into the cytoplasm of oocytes showing the first polar body after piezo drilling to cut the zona pellucida and penetrate the oolemma. Nonmatured oocytes were further matured, subsequently checked until 24 h after decumulation and injected when a polar body was detected. Only very few oocytes matured after removal of cumulus cells and almost all of them remained uncleaved after ICSI. Moreover, the few cleaved ones always failed to develop into viable embryos; therefore, in the data analysis, these oocytes were considered as not mature.

Injected oocytes (day 0) were cultured at 37.5°C in a humidified atmosphere at 5% CO_2_ and 5% O_2_ in modified SOF medium supplemented with BSA and MEM amino acids (Lazzari *et al.* 2002) using a GERI time-lapse incubator (Merck KGaA). Cleavage was assessed at day 2 post injection. Half of the medium was first changed on day 4 after ICSI and then every 48 h until freezing.

The time-lapse images were taken every 5 min on 11 different focal planes and the resulting videos were analyzed with the Geri Assess software for further studies.

Due to the low quality and fertilization ability of the NWR semen, determined in preliminary tests on swine oocytes as described in Hildebrandt *et al.* (2018a), the oocytes injected with NWR spermatozoa were electrically activated within 1 h after ICSI. Oocytes were washed in a 0.3 M mannitol solution supplemented with calcium and magnesium (0.05 mM and 0.1 mM, respectively), transferred into a cell fusion machine chamber (SpyZot, LPS electronics, Cremona, Italy), subjected to two DC pulses of 1.5 kV/cm for 30 µs, and returned to the culture medium in the incubator.

### Blastocyst cryopreservation

Embryos were cryopreserved via slow freezing. Cleaved oocytes that developed into blastocysts were evaluated for freezing from day 7 onward. When an ICM and a clear trophoblast layer of cells were visible and the blastocyst had begun to expand with consequent thinning of the zona pellucida, they were equilibrated with the cryoprotectant for 5 min in 5% glycerol in H-SOF and then for 20 min in 10% glycerol at room temperature, during which the embryos were loaded into straws. The sealed straws were plunged in a methanol bath freezing machine (Biocool IV, FTS systems) precooled at −6°C. After 5 min, seeding was induced with a lN_2_-cooled forceps. Ten minutes after seeding, the freezing program started with a cooling ramp of 0.5°C/min until reaching −32°C. Then the straws were plunged into lN_2_ and stored.

### Hormonal status and cycling activity

Hormonal status was assessed for 21 OPU procedures in 12 SWR females in European zoos (average age: 16.5 ± 6.6 years, range: 7.2–24.3 years). Serum progesterone (chemiluminescent immunoassay, CLIA), serum 17ß-estradiol (radioimmunoassay, RIA), and additionally in 4 OPU procedures in 3 individuals (13–24 years), anti-Muellerian hormone (AMH; enzyme-linked immunosorbent assay, ELISA) was measured (equine protocol) by a commercial laboratory (Idexx Laboratories, Vet Med Labor GmbH, Germany).

Cycling activity was determined based on behavioral observations or fecal hormonal analyses by the respective zoological facilities. The presence of corpora haemorraghica (CH) or corpora lutea (CL) on the ovaries was documented via ultrasound during OPU procedures.

#### Definition of terms

Oocyte recovery rate denotes the number of retrieved oocytes in relation to a number of punctured follicles during OPU. Embryo success rate refers to the number of blastocyst-stage embryos generated in relation to the number of collected oocytes.

#### Statistical analysis

Data were statistically analyzed using R (Version 4.1.2). To test for effects on blastocyst generation of individual age at OPU, subspecies, season, and the year of OPU experience, we fit a generalized linear mixed-effects model (GLMM) with a number of generated blastocysts as response and age, year of experience (1st–8th), season (spring = 1; summer = 2; autumn* = *3; winter = 4), and subspecies (NWR = 1, SWR = 0) as the fixed effects terms and individual as random-effects term, assuming a Poisson distribution. We used the glmer function of the R-package lme4 in R version 4.1.2 (R Core Team 2022) (full model: glmer (blastocysts ~ age + year + season + subspecies+(1|id), family = “poisson”)). We checked the assumption of normally distributed residuals by visually inspecting the quantile–quantileplot and observed no obvious deviations.

For further analysis, data were tested using the Wilcoxon rank sum test (WRST) or the Pearson’s product–moment correlation or Pearson’s correlation (PC) using the cor.test function (Best & Roberts 1975, Hollander *et al.* 2014). Significance threshold was set at 0.05. Data are presented as value ± s.d.

## Results

We analyze 65 OPU–IVF procedures performed between 2015 and 2022 according to donor reproductive health; number of total and punctured ovarian follicles; follicle diameters; number of recovered and of degenerated, matured, nonmatured oocytes, second meiosis metaphase (MII)-stage oocytes; cleavage; blastocyst generation; embryo success rate (i.e. blastocyst-stage embryos per number of collected oocytes); age; season; subspecies (SWR vs NWR); origin (captive vs wild-caught); serum progesterone and estradiol levels; presence of CL or CH; and hormonal stimulation protocol (data summary: Table 1; statistics: Table 2).

**Table 1:** Summary of the results of 65 IVF procedures in 22 white rhinoceros oocyte (WRO) donor females, 20 Southern white rhinos (SWR) and 2 Northern white rhinos (NWR), between 2015 and 2022.

	Mean ± s.d.	Max	Min	WRO, n	Total NWR, *n*	Total SWR, *n*	NWR + SWR, *n*
*n*				22	2	20	22
OPU^*^				61	14	51	65
Age at OPU, years	20.0 ± 8.2	31.4	7.2	65			
Origin of donor female^†^	0.7 ± 0.5	1.0	0.0	65			
Ovarian cycling activity^‡^	0.4 ± 0.4	1.0	0.0	65			
Follicles^§^*n*	23.2 ± 14.5	48	0	65	464	1041	1505
Follicles punctured during OPU, *n*	18.0 ± 12.6	46	0	65	384	787	1171
Oocytes retrieved during OPU, *n*	6.2 ± 6.1	23	0	65	156	246	402
Nonmatured oocytes, *n*	3.5 ± 3.7	16	0	55	83	107	190
Degenerate oocytes, *n*	1.0 ± 1.7	8	0	55	9	44	53
MII-stage oocytes, *n*	2.7 ± 2.8	12	0	55	64	86	150
Cleaved embryos, *n*	1.4 ± 1.7	6	0	55	30	45	75
Blastocysts cryopreserved, *n*	0.8 ± 1.2	5	0	65	22	29	51
Maximal follicle diameter, mm	25 ± 7	34	13	55			
Minimal follicle diameter, mm	7 ± 3	14	4	55			
Average follicle diameter, mm	15 ± 3	22	9	55			
s.d. of follicle diameter	6 ± 3	10	2	54			
Serum progesterone, ng/mL	1.1 ± 1.2	3.7	0.2	21			
Serum 17-β estradiol, pg/mL	21.9 ± 12.3	50.8	9.3	21			
CH/CL	0.3 ± 0.5	1	0	65	3	17	20
Recovered oocytes^║^	0.3 ± 0.2	0.7	0.0	59			
Successfully generated blastocytes^¶^	0.1 ± 0.2	0.7	0.0	55			

^*^NWR/SWR: subspecies (1 = NWR, 0 = SWR; 20 % of OPU were performed in NWR); ^†^origin of the donor female (1 = wild-born, 0 = captive-born); ^‡^ovarian cycling activity (1 = cycling, 0.5 = irregularly cycling, 0 = noncycling); ^§^number of follicles as counted by ultrasound at the beginning of OPU; ^║^number of recovered oocytes divided by number of punctured follicles; ^¶^number of successfully generated blastocysts divided by number of oocytes.

CH/CL, presence (=1) or absence (=0) of a corpus hemorrhagicum or corpus luteum; max, maximal value; min, minimal value; OPU, ovum pick-up.

**Table 2:** Blastocyst generation success of 65 IVF procedures in 22 white rhinoceroses from 2015 to 2022 – generalized linear mixed-effects model (GLMM) results for effects of age, subspecies, study year, and time of the year. Data were statistically analyzed using R (Version 4.1.2). To test for effects on blastocyst generation of individual age at OPU, subspecies, season, and of year of OPU experience, we fit a GLMM with number of generated blastocysts as response and age, year of experience (1st–8th), season (spring = 1; summer = 2; autumn* = *3; winter = 4), and subspecies (NWR = 1, SWR = 0) as the fixed-effects terms and individual as random-effects term, assuming a Poisson distribution. We used the glmer function of the R-package lme4 in R version 4.1.2 (R Core Team 2022) (full model: glmer (blastocysts ~ age + year + season + subspecies + (1|id), family = ’poisson’)). We checked the assumption of normally distributed residuals by visually inspecting the quantile–quantile plot and observed no obvious deviations.

	Fixed effects			
	β-estimate	s.e.	*Z* value	Pr(>|z|)
Intercept	0.42	0.82	0.51	0.61
Age	−0.11	0.04	−2.46	0.014^*^
Year	0.29	0.10	2.76	0.0057^†^
Season	−0.33	0.14	−2.41	0.016^*^
Subspecies	1.10	0.68	1.63	0.10

^*/†^Results indicate significant effects of age, study year, and season, respectively, on the successful generation of blastocysts across the examined 65 OPU procedures.

### Animals and health effects

The study comprises 22 female white rhinoceroses, 20 SWR and 2 NWR.

**Figure 1:**
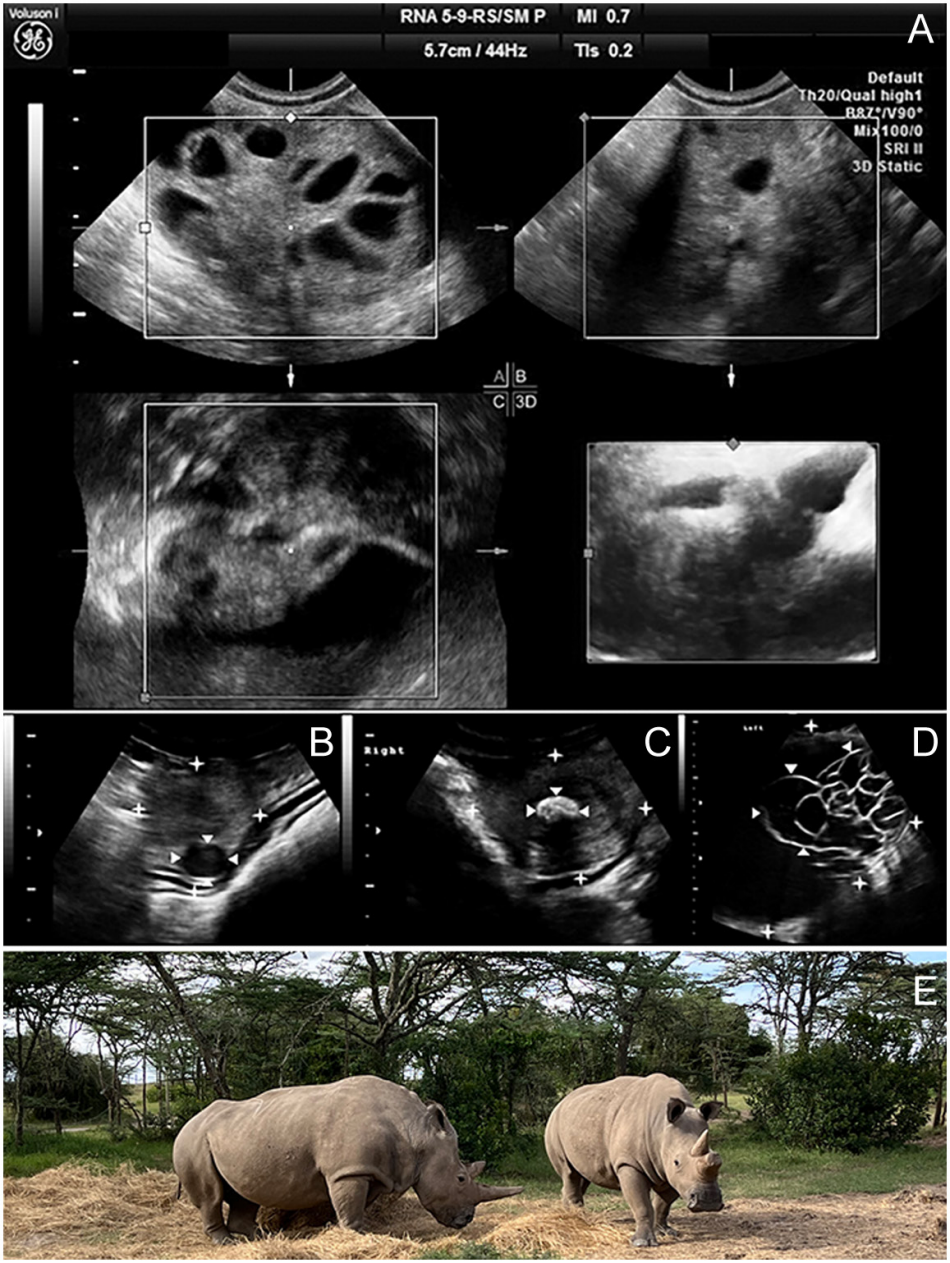
(A) Ovarian health after repeated OPUs in the Northern white rhino (NWR) Fatu, and (B–D) preexisting pathologies of the NWR Najin’s reproductive tract, first detected in 2014, contributed to the decision to retire her as an oocyte donor in 2021. (A) A 3D tomographic ultrasound image of the ovary of the NWR Fatu immediately before the eighth OPU over the course of 29 months. Both follicles and parenchyma show no indications of compromised ovarian health or function, despite performing an OPU on average every 3 months. The image was generated using a portable 3D/4D Voluson I ultrasound unit (GE Healthcare). (B) One of multiple small (≤1.5 cm) leiomyomata (arrows) in both of Najin’s uterine horns (stars); ultrasound image (Voluson I, RNA5-9-D 3D/4D Convex Probe, GE Healthcare, Germany). (C) Uterine adenoma (1.6 cm; arrows) in Najin’s right uterine horn (stars). (D) Large neoplastic formation (stars) of heterogeneous and cystic (arrows) texture on the adnexus of Najin’s left ovary of 176 mm × 114 mm; ultrasound images (Voluson i, RNA5-9-D 3D/4D Convex Probe, GE Healthcare). (E) The last two Northern white rhinoceroses, females Najin (left) and Fatu (right) at Ol Pejeta conservancy. Photo: Susanne Holtze.

Even repeated OPUs yielded no indications of adverse effects on general and reproductive health, such as inflammation, pathological alterations, or declining response to ovarian stimulation (Fig. 1A). After aspiration of thick-walled ovarian cysts in two SWR during OPU, follicle numbers in response to hormonal stimulation increased (1 follicle in the first vs 5.5 ± 2.6 in four subsequent OPUs, and 5.0 ± 1.4 follicles in the first two vs 11.0 ± 1.4 in two subsequent OPUs, respectively). The pathological cystic ovarian structure of 1 NWR (Fatu) undergoing 10 OPUs over 3 years regressed from ~50 mm in diameter in 2019 to ~15 mm in 2022.

Two cycling SWR females are currently pregnant after natural mating, following four and one and five and two OPUs and ET attempts, respectively. One formerly noncycling SWR resumed cycling, became pregnant, and delivered a healthy male calf subsequent to two successful OPUs and one ET. In two SWR, assumed infertile, procedures were discontinued after unexpected ultrasonographic visualization of pregnancy during the scheduled OPU: one with a long history of mating intervals of 4–6 months was early pregnant (day ~25) and failed to maintain pregnancy. The second female was in her fifth gestational month and gave birth to a healthy female calf.

Despite preceding hormonal stimulation, no physiological follicular structures were ultrasonographically detectable in three SWR aged 25.8, 36.7, and 45.0 years, respectively, prompting their exclusion from the program. No drug resistance was observed; anesthetic dosages remained constant across repeated procedures.

Reproductive and general health-related pathologies were frequently detected in the study cohort using ultrasound. Pathologies prevailed in older, non-reproductive females, affecting 72% of our study population (14 of 20 SWR and both NWR). Sometimes several alterations co-occurred in one individual, comprising endometrial (8 SWR), oviductal (2 SWR), and ovarian (3 SWR, 1 NWR) cysts, cysts in the ovarian adnexal region (2 SWR, 1 NWR), degenerated endometrium (3 SWR, 2 NWR), moderate ascites (2 SWR, 2 NWR), and reactive lymph nodes (1 SWR) and kidney cysts (one SWR).

Ovarian tumors were shown in 2 individuals (1 SWR, 1 NWR) as irregular masses of 121 mm × 73 mm and 176 mm × 114 mm, with heterogeneous and cystic appearance, respectively. In the NWR (Najin, Fig. 1B, C, and D), moderate progression was observed over the course of 8 years. The SWR was examined only once. Both individuals were subsequently retired from the program.

### Oocyte recovery and *in vitro* preimplantation development

**Figure 2:**
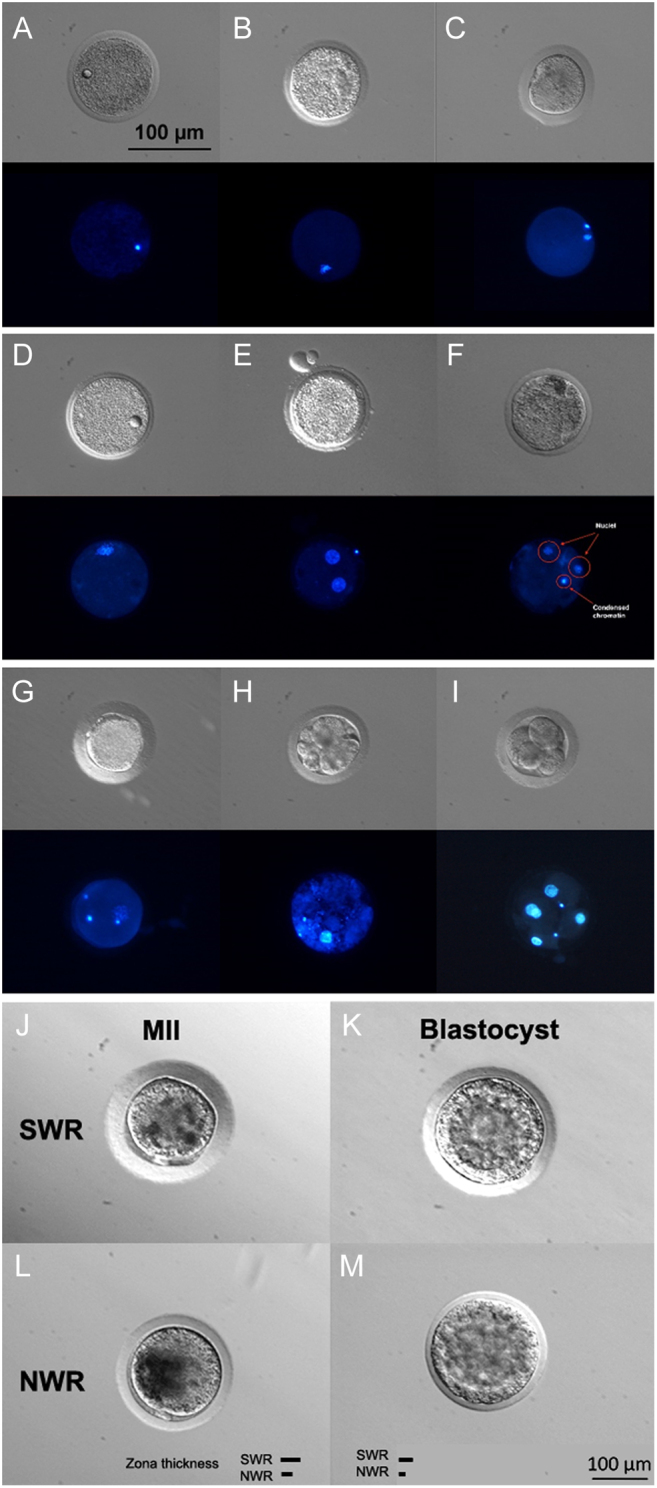
Stages of embryo oocyte maturation and embryo development in SWR and NWR. (A–I) Microscopic images in bright-field with corresponding Hoechst stain and (J–M) comparison of SWR and NWR MII oocyte and blastocyst morphology in bright field. (A) SWR oocyte with a lipidic droplet and germinal vesicle. The scale bar applies to images A–I. (B) SWR nonmatured MI oocyte. (C) SWR oocyte late MII. (D) SWR oocyte with lipidic droplet in telophase II. (E) NWR in MI stage 72 h after ICSI uncleaved with two pronuclei (PNs) in the absence of extruding polar body (PB), probably induced by the electrical activation. (F) NWR oocyte without cumulus after IVM fragmented into two nuclei and one spot of condensed chromatin, probably due to a spontaneous activation of the oocyte. (G) SWR uncleaved oocyte with 2 PB, 1 PN, and sperm head (SH) without decondensation. (H) SWR degenerated embryo day 4. (I) SWR five-cell embryo. (J) SWR MII oocyte with PB. (K) SWR blastocyst. (L) NWR oocyte with PB. (M) NWR blastocyst. The scale bar applies to images J–M. Note the lesser thickness of the zona pellucida of NWR compared to SWR.

**Figure 3:**
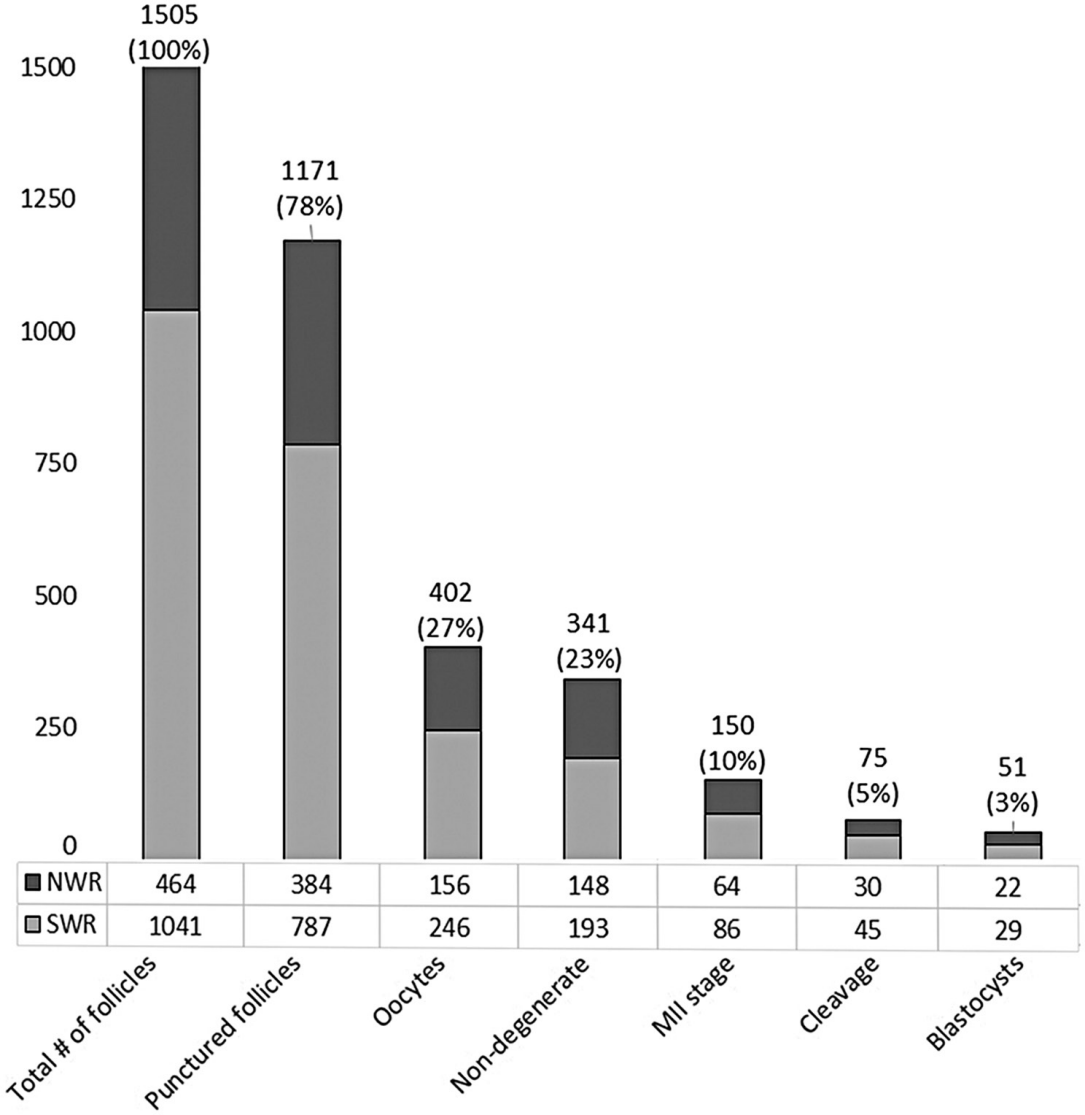
Success of IVF steps in white rhinoceroses during 65 ovum pick-up (OPU) procedures, divided by NWR and SWR. Total counts and success rates for the individual steps of OPU and *in vitro* preimplantation development are given, i.e. the total number of follicles as detected by ovarian ultrasonography, punctured and aspirated follicles, and retrieved oocytes as well as numbers of nondegenerate, MII-stage and cleaved oocytes and the resulting success in terms of blastocyst generation. Percentages are given in relation to the total number of follicles as detected by ultrasound. Altogether 51 blastocysts were generated, 22 pure NWR blastocysts, and 29 blastocysts derived from SWR oocytes, comprising 19 pure SWR blastocysts and 10 SWR/NWR hybrids.

Altogether, in 65 OPU procedures, 1505 ovarian follicles were counted via transrectal ultrasound, averaging, 23.2 ± 14.5 follicles per hormonally stimulated cycle. Of those, 1171 follicles, i.e. 18.0 ± 12.6 per OPU, were punctured, flushed, and aspirated (patent: Hildebrandt *et al.* 2018*b*). Nonpunctured follicles (*n = *334) were either too small (≤3 mm), too close to large blood vessels, or too distant from the transrectally positioned OPU device. In the aspirated fluid, we retrieved on average 6.2 ± 6.1 oocytes per OPU. Of the recovered 402 oocytes (34.3%), nine were clearly dead or lost during packaging and processing. Of the 393 laboratory-cultured oocytes, 53 degenerated during maturation (13.5%), while 340 underwent *in vitro* maturation. Of these, 190 failed to mature (48.3%), while 150 reached MII stage (38.2%). Oocytes that failed to reach MII stage 24 h after decumulation were considered unsuitable for fertilization and were stained to assess chromatin status. MII oocytes were injected with a single sperm cell using piezo-ICSI, which resulted in 75 cleaved embryos (19.1%) and 51 blastocysts (12.9%; 22 NWR, 19 SWR and 10 SWR/NWR hybrids) that were subsequently cryopreserved (Figs. 2 and 3; Videos 1 and 2). Thus, 12.7% of all oocytes and 68.0% of all successfully cleaved zygotes reached blastocyst stage. The number of recovered oocytes per OPU significantly correlated with the number of resulting blastocysts (*P* ≤ 4.6 × 10^−10^, *t* = 7.36, *df* = 63, cor = 0.68, Pearson’s correlation (PC)).

Video 1 SWR embryo development. Time-lapse video of the development of a SWR embryo from fertilized oocyte, through cleavage to morula and blastocyst stage (GERI time-lapse incubator, Merck KGaA, Darmstadt, Germany). The time-lapse images were taken every 5 minutes on 11 different focal planes. This video (https://doi.org/10.1530/REP-23-0087.
Download Video 1

Video 2 NWR embryo development. Time-lapse video of the development of a NWR embryo from fertilized oocyte, through cleavage to morula and blastocyst stage (GERI time-lapse incubator, Merck KGaA, Darmstadt, Germany). The time-lapse images were taken every 5 minutes on 11 different focal planes. This video (https://doi.org/10.1530/REP-23-0087.
Download Video 2

Since the generation of the very first *in vitro* SWR blastocysts, the embryo success rate rose to 14.5%, averaging 1.0 ± 1.3 blastocysts per OPU.

### Embryos

The 51 generated and cryopreserved blastocysts comprise 19 pure SWR, 22 pure NWR, and 10 hybrid (SWR oocyte × NWR sperm) embryos. *In vitro* embryo development is shown in Video 1 (SWR) and Video 2 (NWR). So far, 15 blastocysts were used for ET (13 pure SWR and 2 hybrids – ongoing research; no resulting birth yet), 4 for ESC derivation (2 successfully for pure SWR-ESC; 2 unsuccessfully for hybrid SWR/NWR-ESC), and 1 pure SWR embryo for a successful post-thawing vitality test. Currently, 3 pure SWR, 6 hybrid, and 22 pure NWR blastocysts remain cryopreserved for future ET or for ESC generation (Fig. 2K and M).

### IVF program – progression over time

**Figure 4:**
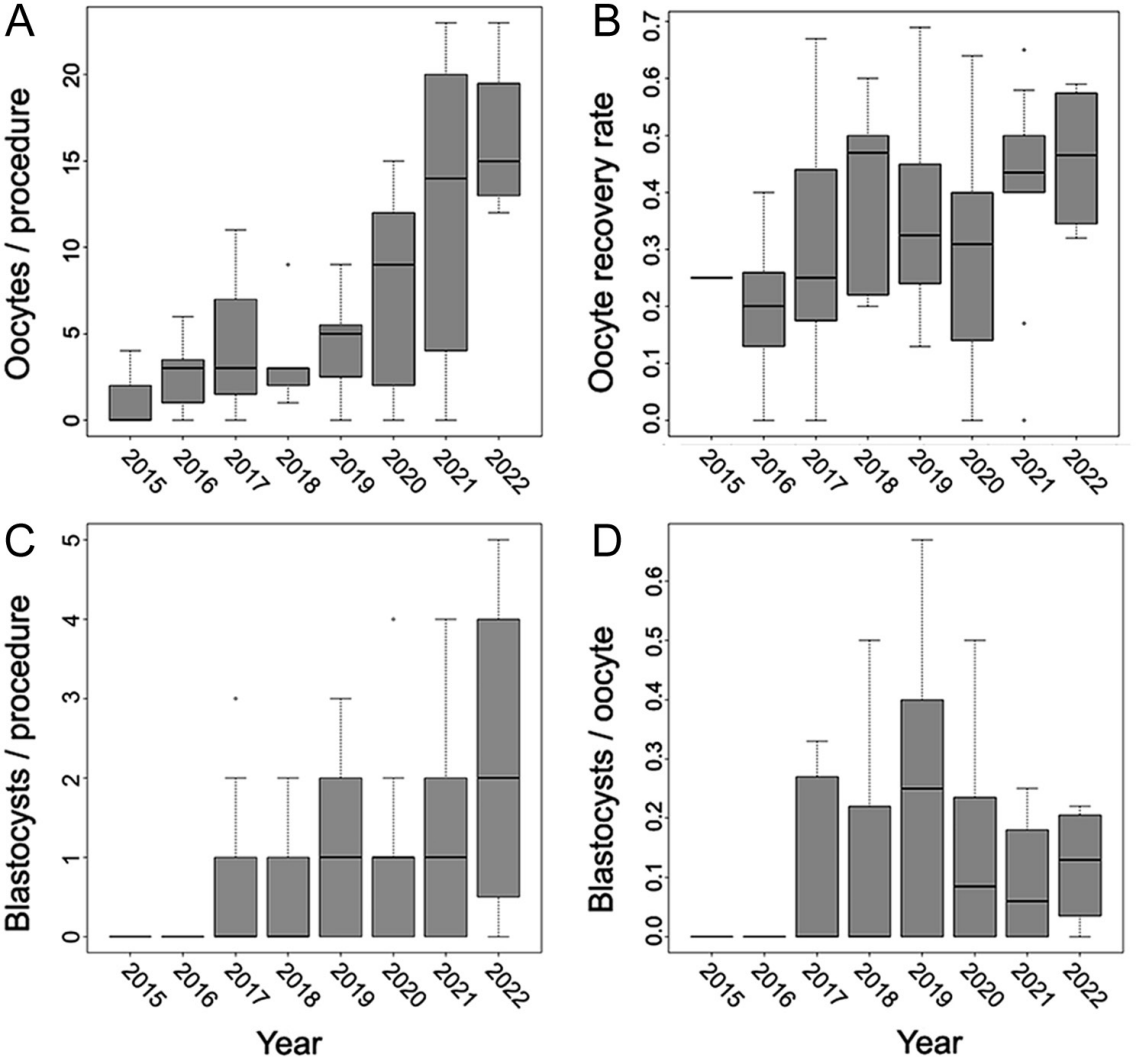
Success of the white rhinoceros IVF program over the course of 8 years (65 procedures from 2015–2022). There is an increase in the (A) number of collected oocytes per ovum pick-up (OPU) procedure, (B) in oocyte recovery rate (oocytes retrieved per punctured follicle), and (C) in blastocysts generated per OPU procedure, as well as an increase followed by a mild decrease from 2019–2022 in (D) blastocyst-stage embryos generated in relation to oocyte numbers for both subspecies, SWR and NWR. This decrease may be due to improved follicle flushing which enhances retrieval of also more immature oocytes with lower developmental capacity. Nevertheless, the ratio of blastocysts per OPU increased over the study period. The central line represents the median, box height the middle 50% of the data, and the box edges the 25th and 75th percentiles. The whiskers represent 1.5 × IQR (interquartile range).

Over the 8-year study period, the efficiency improved across all steps of the IVF process (Tables 2 and 4). Embryo success rate increased from 2015 to 2019, followed by a slight decline from 2019 to 2022 (Fig. 4).

### Age

**Figure 5:**
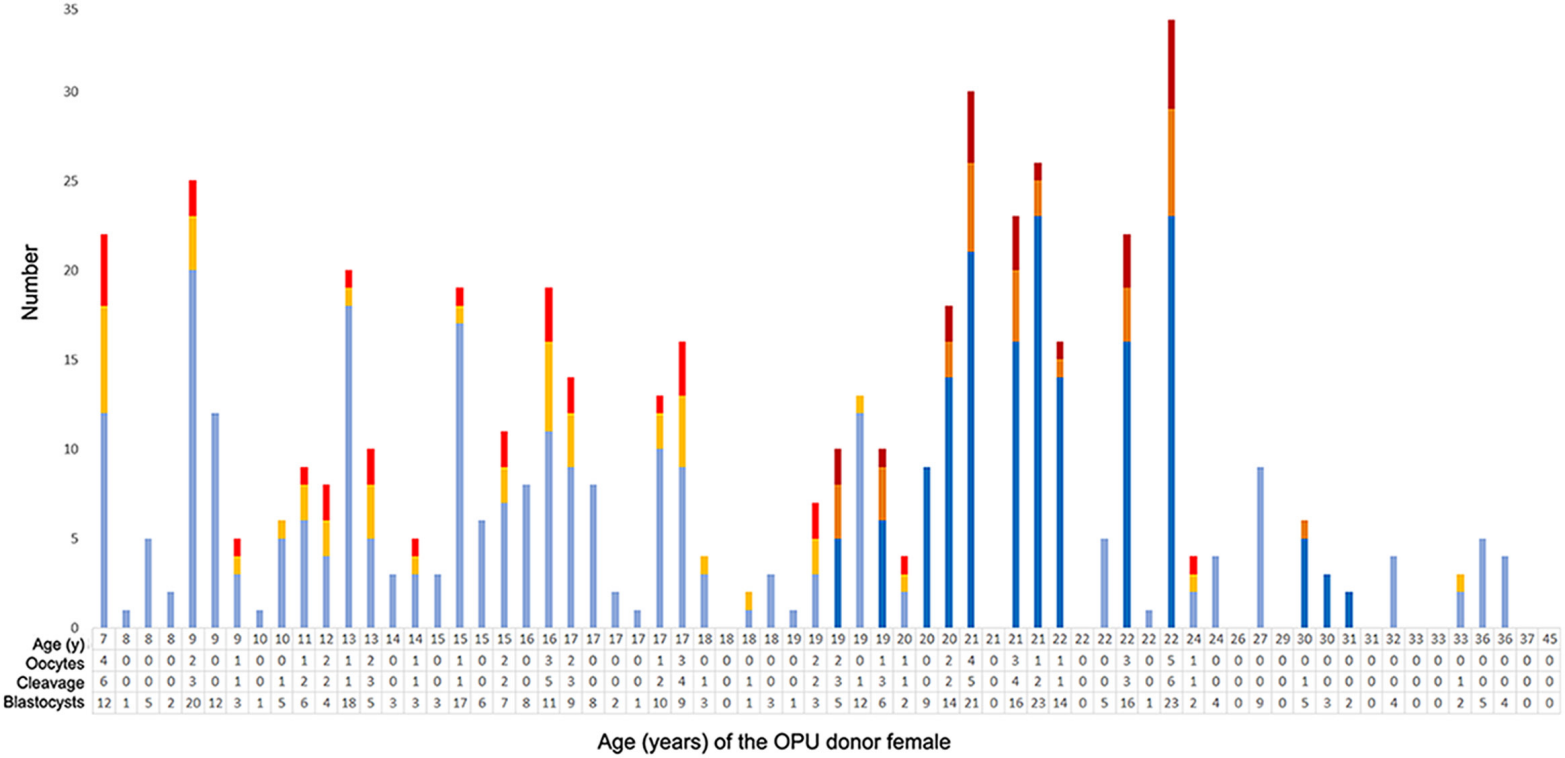
Outcome of 65 IVF procedures relative to oocyte donor age in 22 white rhinoceroses (WR), 20 SWR and 2 NWR over an 8-year study period. Several oocyte donors were subjected to OPU repeatedly (up to 10 times) and therefore may appear several times in the graph. Success of oocyte retrieval (oocytes, light/dark blue), cleavage-stage embryos (cleavage, yellow/orange), and successful *in vitro* blastocyst generation (embryos, light/dark red) is given as a function of age of female WR oocyte donors. Light colors (light blue/yellow/light red) represent SWR (*n = *20), dark colors (dark blue/orange/dark red) represent NWR (*n = *2). Note that no viable blastocysts were generated in any WR oocyte donor female older than 24 years.

Age significantly inversely correlated with blastocyst generation success (*P* ≤ 0.014, GLMM; Table 2). The youngest, 7-year-old donor female at 1 year before onset of ovarian cyclic activity achieved the highest blastocyst-to-oocyte-ratio in any SWR OPU (4 blastocysts out of 12 oocytes). Cleavage was observed up to a donor age of 33 years, but no viable blastocyst was generated beyond 24 years. In females ≥25 years, no oocytes were retrieved in approximately half of the OPUs (7 of 15), with a total absence of follicles in three individuals; in contrast, only three of 51 procedures (6%) failed to yield oocytes in the age group ≤24 years (Fig. 5).

### Seasonality

OPU outcome was highest during the months corresponding to European spring/summer and the main rainy season in Kenya (*P* ≤ 0.016, GLMM; Table 2).

**Figure 6:**
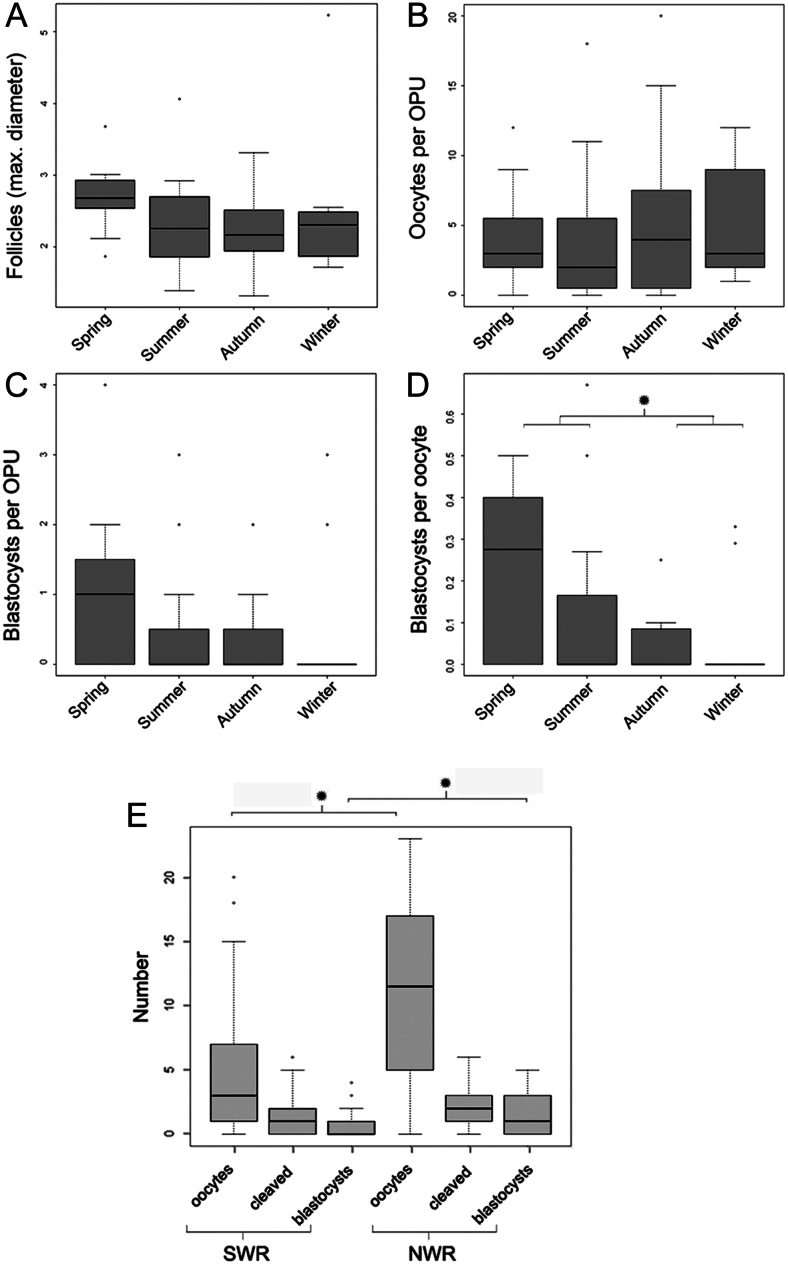
Influence of season on the IVF success of SWR and of SWR compared to NWR IVF success. Seasonal influence was analyzed for female SWR alone due to climatic differences between both subspecies populations for all OPU procedures (*n = *51) in European zoos across the different seasons: spring (March–May), summer (June–August), autumn (September–November), and winter (December–February); ^*^indicates statistical significance. There is a slight seasonal influence regarding (A) maximal follicle diameters, and (C) blastocyst-stage embryos. (D) A significantly higher number of blastocyst-stage embryos was generated per oocyte in spring and summer compared to autumn and winter procedures (*P* ≤ 0.047, *W* = 291,* df* = 20, unpaired two-samples Wilcoxon rank sum test). (E) Number of retrieved oocytes, cleavage and blastocyst-stage embryos generated across all OPU procedures (*n = *65) are higher for NWR compared to SWR, reaching statistical significance with respect to the number of oocytes recovered per procedure (*P* ≤ 0.0034, *W* = 174, Wilcoxon rank sum test) and for blastocyst generation success (*P* ≤ 0.016,* W* = 223, Wilcoxon rank sum test). The central line represents the median, box height the middle 50% of the data and the box edges the 25th and 75th percentiles. The whiskers represent 1.5 × IQR (interquartile range).

Seasonal effects were assessed for SWR alone, due to expected different seasonal effects in the northern hemisphere compared to near equatorial NWR for which, additionally, OPU data are limited to only two individuals. While systematic seasonal differences in follicle numbers, recovery rate, and oocytes retrieved per OPU (Fig. 6B) were absent, maximal follicle diameters were slightly larger (2.50 ± 0.64 vs 2.34 ± 0.79; Fig. 6A), and blastocyst per oocyte rate was significantly higher in spring and summer compared to autumn and winter procedures (Fig. 6D; *P* ≤ 0.047, *W* = 291, *df* = 20, unpaired two-samples Wilcoxon rank sum test (WRST)).

### Subspecies

Compared to SWR, success in NWR oocyte yield (11.1 ± 7.8 vs 4.6 ± 4.8) and blastocyst generation per OPU (1.6 ± 1.7 vs 0.6 ± 1.0) were significantly higher (Fig. 6E). This reached statistical significance with respect to the number of oocytes recovered per procedure (*P* ≤ 0.0034, *W* = 174, WRST), number of oocytes reaching MII stage (*P* ≤ 0.0030, *W* = 125.5, WRST), and for blastocyst generation success (*P ≤ 0*0.016, W=223, WRST); however, it failed to reach statistical significance in the general linear model (*P ≤ 0*0.10, GLMM, Table 2).

### Origin of individuals

Wild-born individuals (*n = *10; average age at OPU 20.2 ± 8.8 years) were more likely to show cycling activity (50.0% vs 31.2%) compared to captive-born ones (*n = *12; average age at OPU 20.1 ± 8.1 years) (Tubbs *et al.* 2016). However, SWR cleavage rate (*P* ≤ 0.0028, *t *= 3.14, *df* = 53, PC) and blastocysts generated per OPU (*P* ≤ 0.014, *t* = 2.58, *df* = 40, PC) were significantly lower in wild-born compared to captive-born individuals.

### Hormonal status and cyclicity

Hormonal status was determined for 21 OPUs in 12 SWR females in European zoos, 55% of which showed cycling activity. Serum estradiol levels at OPU averaged 21.9 ± 12.3 pg/mL and serum progesterone levels 1.1 ± 1.2 ng/mL. Statistically, across all procedures, a positive correlation was detected between serum progesterone levels and reported ovarian cyclicity (*P* ≤ 0.015, *t* = 2.67, *df* = 19, PC). Serum progesterone levels also positively correlated with maximal follicle diameter (*P* ≤ 0.0020, *t* = 3.66, *df* = 17, PC). However, no positive correlation of serum progesterone levels was evident with cleavage (*P* ≤ 0.81, *t* = 0.25, *df* = 17, PC) or blastocyst rate (*P* ≤ 0.87, *t* = 1.67, *df* = 19, PC), and no significant correlations with serum estradiol levels were detected.

Ultrasonographic presence of a CL or corpus hemorrhagicum (CH) on the ovaries was evident in 32.3% of all OPU procedures and positively correlated with cycling activity (*P* ≤ 9.89 × 10^−5^, *t* = 4.16, *df* = 62, PC). Cycling activity correlated negatively with age (*P* ≤ 4.74 × 10^−7^, *t* =−5.61,*df* = 63, PC). Neither NWR showed cycling activity during the study period.

Serum AMH values (four measurements in three SWR between 13–24 years) were below detection limit of the equine ELISA (<0.01 ng/mL) although two of the OPUs during which blood samples were taken resulted in the generation of three and one blastocysts, respectively.

### Stimulation protocol

The time interval (2, 3, or 4 days) between the OPU and the last of the three GnRH agonist injections significantly positively correlated with average follicle diameter (*P* ≤ 0.037, *t* = 2.14, *df* = 51, PC). However, data are skewed with the majority of procedures adhering to the 3-day interval.

Average follicle diameter negatively correlated with number of ovarian follicles (*P ≤ *0.0051, *t = *−2.92, *df* = 54, PC), punctured follicles (*P ≤* 0.012, *t = *−2.59,*df* = 54, PC), and recovered oocytes (*P ≤ 0*0.0056, *t = *−2.89, *df* = 54, PC), but it also negatively correlated with the number of oocytes that failed to mature (*P ≤* 0.013, *t = *−2.56, *df* = 50, PC). Overall, no significant correlation was evident between average follicle diameter and number of blastocysts generated per OPU (*P ≤ 0*0.33, *t = *−0.98, *df* = 54, PC) or per oocyte (*P ≤* 0.23, *t = *1.21, *df* = 50, PC).

The single OPU performed without prior hormonal stimulation yielded a relatively high number of follicles and oocytes. Follicle sizes were smaller compared to those during the four stimulated OPU cycles in the same female, whereas blastocyst generation success was comparable between the unstimulated and the four stimulated procedures (Table 3).

**Table 3:** Success of stimulated (*n = *4) versus unstimulated (*n = *1) ovum pick-ups (OPUs) and* in vitro* embryo generation in the same southern white rhinoceros female. Comparison of the success of hormonally stimulated (*n = *4) and unstimulated (*n = *1) OPU cycles in the same naturally cycling female SWR from 2016 to2020. Data are presented as mean ± s.d. The number of ovarian follicles in the OPU without prior hormonal stimulation surprisingly is higher as compared to the respective average of four OPUs in the same female with preceding hormonal stimulation using a GnRH agonist. While follicle diameters are smaller, a higher number of oocytes was recovered from the unstimulated OPU, and similar blastocyst numbers were generated from both, the single unstimulated and stimulated procedures.

	Stimulated OPU	Unstimulated OPU
OPU cycles, n	4	1
Follicles	22.5 ± 3.4	37
Punctured follicles	18.0 ± 3.6	32
Follicle diameter, mm		
Max	28 ± 2	22
Min	6 ± 2	5
Average	17 ± 2	10
Oocytes	4.5 ± 1.7	16
Cleaved	1.8 ± 1.0	1
Blastocysts	1 ± 0.8	1

## Discussion

We examined the effects of OPU and* in vitro* preimplantation development success related to health, age, season, subspecies, captive-born vswild-caught origin, hormonal status, follicle diameters, and hormonal stimulation protocol across 65 procedures in 20 SWR and 2 NWR females from 2015 to 2022. We successfully generated 51 blastocysts and 2 embryonic stem cell lines and currently store 3 SWR, 6 SWR/NWR hybrid, and 22 NWR cryopreserved blastocysts for future embryo transfer.

### Animals and health effects

To address the risks associated with this novel technology in two endangered rhinoceros taxa, particularly regarding the unusual transrectal approach to ovarian puncture due to anatomical constraints, we carefully assessed any potential reproductive and health-related implications of rhino OPU.

There was no evidence for detrimental effects of repeated OPU procedures with prior hormonal stimulation on reproductive health, fertility, cycling activity, ultrasonographically assessed ovarian morphology (Fig. 1), follicle numbers in response to hormonal stimulation, or success across all levels of the IVF program. To the contrary, removal of potentially hormone-active cysts during ovarian puncture, as performed in two females, improved ovarian morphology and increased follicle numbers on the affected ovary in response to hormonal stimulation. This is in accordance with beneficial effects of ovarian cyst aspiration in humans (Rizk *et al.* 1990). Furthermore, the NWR Fatu showed continuous regression of a pathological cystic ovarian structure over the course of ten OPUs within 3 years.

Finally, a formerly noncycling SWR female resumed cycling following two successful OPUs and one unsuccessful ET, conceived naturally, and gave birth to a healthy calf. This indicates a positive influence on cycling activity, while no detrimental effect was observed on procreation: Two SWR are currently naturally pregnant after undergoing repeated OPUs and ETs. In two supposedly infertile SWR females, OPUs were discontinued after unexpected sonographic pregnancy detection. One with a history of irregular mating intervals failed to implant, while the other gave birth to a healthy calf, unaffected by hormonal stimulation and OPU. Our results are consistent with reports of safely performing OPU in livestock during the first three gestational months and in lactating cows (Meintjes *et al.* 1995) or horses (Galli *et al.* 2014).

In heifers, frequent OPUs (twice-weekly for up to 5 weeks) caused merely mild endocrine and morphological alterations, i.e. a minor hardening of the ovaries and thickening of the ovarian tunica albuginea (Petyim *et al.* 2001). As OPU rather enhanced WR reproductive health, this ‘mechanical ovarian cleansing’ could even serve as an optional fertility restoration treatment for sub- or infertile- female rhinoceroses with minor reproductive pathologies.

We found no evidence for adverse effects on general health. The inherent risk of anesthesia can be seen as negligible considering complication-free anesthesia in all 65 reported procedures and in >500 procedures the authors performed in WR for semen collection, reproductive health assessment, or AI.

Ultrasound examination frequently revealed preexisting reproductive tract pathologies in our WR study group, which is explained by both high susceptibility and inclusion criteria defined by the WR European Endangered Species Programme (EEP), focusing on females with a record of failed natural breeding attempts. The resulting bias in our study population toward older, sub- or infertile- females implies that our data may be not representative for WR.

Reproductive pathologies are observed across many mammalian taxa and are well-documented for megaherbivores to correlate with prolonged exposure to oscillating estrogen concentrations during frequent follicular waves. While naturally occurring long periods of absent sexual cycling activity with concomitant rise in progestin during pregnancy and lactation have a protective effect on the reproductive tract, frequent futile cycles facilitate reproductive pathologies. This vicious circle results in reduced fertility and, eventually, irreversible acyclicity, prematurely terminating the reproductive lifespan. This phenomenon was termed ‘asymmetric reproductive aging’ (Hildebrandt & Göritz 1998, Hildebrandt *et al.* 2000, Hermes *et al.* 2004, 2006). Associated pathologies comprise cystic endometrial and ovarian degeneration, and uterine and ovarian tumors, mostly leiomyomata (Hermes *et al.* 2006), consistent with our observations.

Two WR females showed neoplastic structures, among them the older NWR Najin (Fig. 1B, C and D). Her ovarian tumor steadily progressed since its detection in 2014, reaching a dimension that finally impaired OPU in the respective ovary. Among other considerations, the absence of successful blastocyst-stage embryo generation of altogether ten oocytes obtained during four OPUs led to her retirement as oocyte donor (https://www.izw-berlin.de/files/biorescue/FINAL_Report_Najin_October_2021.pdf). The decision was based on a thorough ethical assessment, involving all relevant aspects and stakeholders. Alongside shifting the boundary of available conservation approaches, the authors implemented a holistic ethical framework for embedding each procedure into the larger context of individual welfare and preventing extinction (de Mori *et al.* 2021, Biasetti *et al.* 2022).

In summary, using OPU as a new reproductive management strategy in affected individuals may not only reduce estrogen exposure through follicle aspiration, halting progression, but also include otherwise sterile individuals into the future gene pool. For individuals with preexisting ovarian pathology susceptible to endocrine factors, hormonal stimulation is contraindicated. Here, unstimulated OPU is a viable alternative: in a single tested case, it yielded comparable blastocyst generation success compared to stimulated OPUs of the same SWR donor (Table 3).

### Oocyte recovery and *in vitro* preimplantation development

Notwithstanding the fertility-compromised study cohort, our success rates of oocyte recovery and IVF at Avantea laboratory, the only laboratory so far to reportedly generate blastocyst-stage rhinoceros embryos, are comparable to those achieved in horses (Lazzari *et al.* 2020) and humans yet inferior to pigs (Yoshioka *et al.* 2020) and cattle (Galli *et al.* 2014, Watanabe *et al.* 2017). IVF protocols differ across taxa, compromising direct comparisons. Unlike in humans, rhinoceros and horse oocytes are harvested immature. For prior stimulation of follicle development, a slow-release GnRH agonist is employed in rhinoceroses, while in pigs, porcine follicle-stimulating hormone (Yoshioka *et al.* 2020), in humans a gonadotropin protocol combining a GnRH agonist or antagonist, followed by human chorionic gonadotropin administration (Bosch *et al.* 2020), and in cattle a gonadotropin with or without progesterone (Galli *et al.* 2014) are employed. In mares, oocytes are collected unstimulated. In rhinoceroses, for safety, recumbent anesthesia is essential, whereas in cattle and horses standing sedation with spinal epidural anesthesia is sufficient (Lazzari *et al.* 2021). Further logistic and procedural differences are dictated by donor size and anatomy (Galli *et al.* 2014). Uniquely in WR, a transvaginal approach is unachievable, rendering transrectal follicle puncture the only viable option to reach the ovaries (Hildebrandt *et al.* 2018*a*,*b*).

The relatively low oocyte recovery rate compared to other species is explained by the 85° angle of the needle which impairs needle rotation commonly employed for scraping the inner follicular wall. Furthermore, the long tubing (~2.5 m) as well as collecting immature COCs impair flushing and aspiration. Finally, domestic species have long been selected for fertility, whereas – besides our study population being reproductively impaired – rhinoceroses produce a single offspring every ~2 years.

The complete absence of physiological follicles following hormonal stimulation as observed in three individuals may be age- or health-related or due to incomplete injection of the highly viscous GnRH agonist through the thick cutis.

### IVF program – progression over time

Number of oocytes collected per procedure, oocyte retrieval rate, and embryo success rate considerably increased over time (Fig. 4, Tables 2 and 4) due to (i) technical optimization, (ii) improved team performance, and (iii) slightly beneficial effects of repeated OPUs on donor reproductive health. However, SWR embryo success rate slightly decreased from 2019 to 2022 possibly due to improved follicle flushing (Fig. 4A and B) or enhanced recovery of smaller, immature oocytes with lower developmental capacity. Overall success, i.e. blastocysts generated per OPU, nevertheless increased.

**Table 4:** Statistical analysis of the success of the different IVF steps in white rhinoceros females (*n = *22) over the course of 8 years.

	Pearson’s correlation^*^		
	*P*	*t*-value	*df*^†^
Total number of follicles per procedure	≤0.003	3.1	63
Ratio of punctured follicles per total follicles	≤0.00004	4.4	63
Average number of oocytes retrieved per OPU procedure	≤0.0000001	6.0	63
Oocyte recovery rate	≤0.02	2.5	57
Average number of blastocysts generated per procedure	≤0.0003	3.9	63

^*^Pearson’s correlation as calculated using R (Version 4.1.2); ^†^the study data comprise a total of 65 procedures in 22 white rhinoceros females.

### Age

Donor age is decisive for IVF success (Fig. 5). In our study group, no blastocyst was generated of a donor female ≥25 years. This implicates excluding old individuals from OPU programs given the resource-intensive procedure and benefit-relation (de Mori *et al.* 2021). It further implies that IVF success of the last female NWR oocyte donor Fatu (22 years) may soon cease, although optimal nutrition, physical exercise, Kenyan climate, semi-wild husbandry, and possibly subspecies-related factors could prolong this period.

Overall age-related fertility decline is found in many species, e.g. horses, the closest domestic SWR relative (Catandi *et al.* 2021), and in humans due to oocyte atresia and meiotic spindle degradation, enhancing aneuploidy risk (Owen & Sparzak 2021). Increased WR reproductive pathology and decreased ovarian activity (Hildebrandt & Göritz 1995, Hermes *et al.* 2006) contribute to the observed fertility decline. However, SWR females in North American zoos reportedly gave birth up to an age of 43 years following natural mating (Tubbs *et al.* 2016), indicating large reproductive lifespan variability.

### Seasonality

WR are considered aseasonal breeders although birth numbers of zoo-kept individuals in the northern hemisphere slightly increase in autumn and decrease in spring, corresponding to reduced conception rates in winter compared to summer (Radeke-Auer *et al.* 2022). Based on Kenya Wildlife Service reports of OPC and surrounding national parks, wild SWR birthing rates peak around February. No photoperiodic signal appears to be involved in preferred mating times in the wild, but rather conception is suppressed during resource scarcity, implying the observed pattern in captivity to be husbandry related. Possible explanations are seasonal food quality fluctuations or increased indoor confinement due to cold weather, reducing mating opportunities (Radeke-Auer *et al.* 2022).

Interestingly, *in vitro* blastocyst generation was significantly more successful in spring compared to winter, although high-quality blastocyst-stage SWR embryos were produced across all four seasons. Follicle and retrieved oocyte numbers were unaffected by seasonal fluctuations. This points to either outside temperature-related effects, e.g. during oocyte handling, or to seasonal fluctuations in oocyte quality. The former is improbable given the presence of suitable heating devices and insulation during all crucial steps of the IVF procedure and continuous logger-based temperature monitoring during transportation. Remarkably, although statistically not significant, maximal follicle diameters in spring and summer were slightly larger compared to autumn and winter (Fig. 6A). This, indeed, suggests seasonal changes in oocyte developmental capacity, possibly based on seasonal variations in nutrition or husbandry.

### Subspecies

Both NWR females are comparably old (Najin 34 and Fatu 23 years), without sexual cycling activity and incapable of reproducing on their own owing to irreversibly damaged Achilles tendons after mating with an overly heavy SWR bull (Najin), and severe endometrial degeneration of unknown origin (Fatu) (Saragusty *et al.* 2016). Fortunately,* in vitro* blastocyst production success of the last remaining NWR oocyte donor Fatu is superior to that of the studied SWR in European zoos (Fig. 6E), rendering IVF the only – yet highly promising – pathway for propagating NWR in the near future. This option, however, is only available thanks to cryopreservation of NWR semen from four different males up to two decades ago. The good response to hormonal stimulation, consistently high numbers of retrieved oocytes and success in generating blastocysts may be of great importance for the fate of the subspecies. A generally large intra-individual variation in follicle numbers exists across different donor females, reported also for cattle (Watanabe *et al.* 2017). This may explain the good results together with other potentially favorable factors, among them Fatu’s good health status due to optimal climate, nutrition, and individualized husbandry in Ol Pejeta, Kenya.

### Origin of individuals

Interestingly, cleavage rates and blastocyst numbers per OPU were higher in captive-born (F1) compared to wild-born SWR donor females (F0), despite being of similar age range. This is unexpected in context of the considerably higher reproductive success reported for F0 females in human care compared to F1 females (Swaisgood *et al.* 2006). This bias, however, has been mainly attributed to higher post-copulatory reproductive failure in F1 (Swaisgood *et al.* 2006), most likely due to social reproductive suppression of the daughters by their commonly co-housed mothers. Based on IVF performance, our findings not only contradict a reduced but rather point toward enhanced fertility of F1 compared to F0 females with regard to oocyte developmental capacity. This challenges the idea of excessive levels of phytoestrogens in captive WR (Tubbs *et al.* 2016, Williams *et al.* 2019), as such would be expected to compromise oocyte quality especially in F1 females.

### Hormonal status and cyclicity

A regular sexual cycle length of 29.6 ± 1.8 days (range: 25–38 days) has been reported for WR, with possible occurrence of prolonged sexual cycles of 65–70 days or acyclicity with ongoing follicular waves (Patton *et al.* 1999). Abnormal ovarian activity patterns increase with both age (Hermes *et al.* 2006) – consistent with the here presented data – and husbandry-related factors, such as small enclosure size, by housing mothers together with their daughters, and by housing with a familiar male (Metrione 2010).

Remarkably, serum progesterone levels, although not directly correlated with blastocyst generation rates, positively correlate with larger maximal follicle diameters. This implies that the timing of hormonal stimulation onset in cycling individuals may influence OPU success.

A valuable marker for predicting ovarian response to hormonal stimulation and oocyte quantity and quality is serum anti-Müllerian hormone (AMH). It is successfully used in human (Iliodromiti *et al.* 2014), bovine (Guerreiro *et al.* 2014), and equine (Papas *et al.* 2021) reproductive medicine. Using horse AMH protocols, SWR serum values, however, consistently remained below detection threshold even in the two younger individuals with good ovarian response (13 and 17 years), of whom blastocysts were successfully generated at the time of blood sampling. Furthermore, shortly before sampling, the 13-year-old female had given birth after natural mating, strongly supporting retained fertility, which should be reflected in measurable AMH values. Whether our assays failed to detect rhinoceros AMH or serum levels in rhinoceroses are generally lower than in horses remains to be clarified. In the absence of a suitable assay or a different biomarker indicating declining female fertility, the most reliable predictors for IVF success remain both age and reproductive tract sonomorphology of the donor female.

### Stimulation protocol

Longer time intervals between the last of three GnRH-agonist injections and subsequent OPU led to slightly larger follicle diameters but also reduced numbers of follicles and recovered oocytes – yet without statistically significant effects on blastocyst generation rate. A shorter protocol is preferred, also considering that prolonged exposure to elevated estrogen levels may favor reproductive tract pathologies. The one reported OPU without prior hormonal stimulation resulted in successful blastocyst generation, implying that unstimulated procedures represent a feasible option for wild rhinoceroses or ones with extensive pathologies.

Large intra-individual differences in response to hormonal stimulation are not attributable to age or health status alone. Possible explanations could be differences in husbandry, mainly regarding diet composition and enclosure size. The decisive influence of diet on rhino fertility and reproduction, especially regarding the amount of phytoestrogens and composition of gut microbiota, has been recently pointed out (Tubbs *et al.* 2016, Williams *et al.* 2019). Another husbandry-related factor concerns enclosure size (Metrione 2010), social environment, and group composition. Limited available space impairing physical exercise favors high body condition scores and potentially enhances social stress, both of which may negatively affect reproductive performance.

## Conclusion

We performed 65 OPU procedures in 22 white rhinoceroses during 8 years with steadily increasing success in generating viable blastocysts. Besides season, donor age is decisive for success of oocyte harvest and* in vitro* embryo production, with an absence in blastocyst generation in females older than 24 years. We found no adverse health effects even of repeated OPUs in the same donor for up to 10 times at a 3-month interval. Contrarily, hormonal stimulation and OPU rather enhanced reproductive health which therefore may be recommended as ‘mechanical ovarian cleansing to restore fertility’ of subfertile WR. We therefore conclude that the described IVF program represents a safe, reliable, and projectable assisted reproductive technology in rhinoceroses. The successful generation of so far 51 blastocysts, 29 of SWR, and 22 of NWR underlines the importance of the technology and its potential to address one of the most pressing global problems: species conservation (Rockström *et al.* 2009). It is our responsibility and in our own interest as humankind to halt and reverse the current dramatic loss of biodiversity. This decline causes an incalculable disturbance of crucial ecosystem services while simultaneously fostering the emergence of novel pathogens (O’Callaghan-Gordo & Antó 2020), which undermines the basis of our very existence (Steffen *et al.* 2015).

## Declaration of interest

The authors declare that there is no conflict of interest that could be perceived as prejudicing the impartiality of the research reported.

## Funding

This study was funded by the BMBF project ‘BioRescue’ (01LC1902A) by the Nadace ČEZ projects PR20/1251 and PR21/46514. Laboratory work at Avantea was, partially, most generously supported by Richard McLellan and by Merck http://dx.doi.org/10.13039/100004334 KGaA, Darmstadt, Germany, with the donation of a GERI time-lapse incubator to Avantea Foundation.

## Author contribution statement

TBH, SH, RH, and FG conceived, designed, and performed all animal experiments, interpreted the data, coordinated the work. TBH and SD received the BioRescue grant (Federal Ministry of Education and Research, Germany, 01LC1902A). TBH holds together with A Schnorrenberg the international patent for the OPU system. JS, IL, DN, PO, LK, DM, SM, and SN prepared and jointly performed the oocyte collections in the northern white rhinoceroses. SC, GL, and CG performed all *in vitro* experiments, collected, and analyzed the laboratory data. SD and KH provided advice for the experimental design of the project. B de M and PB supervised the project in regard to ethical aspects. SH, SC, and AQ analyzed the data. SH wrote the manuscript. TBH, CG, and SC revised the paper. All authors jointly contributed to the final version of the manuscript.
